# Endothelial dysfunction in retinal vessels of hemodialysis patients compared to healthy controls

**DOI:** 10.1038/s41598-024-64581-9

**Published:** 2024-06-17

**Authors:** Roman Günthner, Georg Lorenz, Matthias Christoph Braunisch, Susanne Angermann, Julia Matschkal, Renate Hausinger, Timon Kuchler, Patrizia Glaser, Felix Schicktanz, Bernhard Haller, Uwe Heemann, Lukas Streese, Henner Hanssen, Konstantin Kotliar, Christoph Schmaderer

**Affiliations:** 1 Department of Nephrology, School of Medicine, Klinikum Rechts der Isar, Technical University of Munich, Ismaninger Str. 22, 81675 Munich, Germany; 2 School of Medicine, Klinikum Rechts der Isar, Institute of AI and Informatics in Medicine, Technical University of Munich, Ismaninger Straße 22, 81675 Munich, Germany; 3 Preventive Sports Medicine and Systems Physiology, Department of Sport, Exercise and Health, University of Basel, Basel, Switzerland; 4 Faculty of Health Care, Niederrhein University of Applied Sciences, Krefeld, Germany; 5 Aachen University of Applied Sciences, Heinrich-Mussmann-Str. 1, 52428 Jülich, Germany

**Keywords:** Renal replacement therapy, Arterial stiffening, Vascular diseases

## Abstract

Endothelial dysfunction is a key factor promoting atherosclerosis and cardiovascular complications. Hemodialysis patients typically show various cardiovascular complications and impaired retinal venular dilation has been described as a risk factor for mortality. Non-invasive retinal vessel analysis provides insight into the microvasculature and endothelial function. Static retinal vessel analysis determines arteriolar and venular vessel diameters and dynamic retinal vessel analysis measures microvascular function by flicker-light induced stimulation, which results in physiological dilation of retinal vessels. We measured 220 healthy individuals and compared them to our preexisting cohort of hemodialysis patients (275 for static and 214 for dynamic analysis). Regarding static vessel diameters, hemodialysis patients and healthy individuals did not significantly differ between vessel diameters. Dynamic retinal vessel analysis showed attenuated dilation of the arteriole of hemodialysis patients with 1.6% vs 2.3% in healthy individuals (p = 0.009). Case–control matching for age (mean 65.4 years) did not relevantly diminish the difference. Hemodialysis patients also exhibited reduced venular dilation after matching for age (3.2% vs 3.8%, p = 0.019). Hemodialysis patients showed microvascular dysfunction compared to healthy individuals when using dynamic retinal vessel analysis. Further studies should focus on dynamic retinal vessel analysis which can add insights into the microvascular function and risk factors in multimorbid patients.

## Introduction

Patients with end-stage kidney disease exhibit multiple cardiovascular (CV) comorbidities and increased CV-mortality up to 20 times higher than the general population^1^. Multiple factors are contributing to this highly increased morbidity and mortality. Other than the traditional CV risk factors like arterial hypertension, diabetes mellitus and ventricular hypertrophy, non-traditional risk factors such as uremic toxins, oxidative stress and chronic inflammation have also been the focus of recent research in end-stage kidney disease patients^1,2^. Especially, non-traditional factors contribute to endothelial cell dysfunction (ECD), which is mainly characterized by decreased NO bioavailability^3^. The integrity of the endothelium is crucial for vascular homeostasis and therefore ECD poses one of the key factors for the development of atherosclerosis, which is the driving force for the development of cardiovascular comorbidities^4^.

Measuring ECD in a clinical setting is tricky, because arterioles and venules are not easily accessible. Other methods like flow-mediated vasodilatation, laser doppler flowmetry and venous occlusion plethysmography all focus on indirect assessment of microvascular dysfunction^3^. Retinal vessel analysis provides a convenient tool, which allows direct measurement including functional assessment of small vessels. The additional advantage of retinal vessel analysis is the possibility to separately observe the arteriolar and venular part of the microcirculation. Static retinal vessel analysis (SVA) measures diameters of retinal vessels and calculates an arterial (CRAE) and venous (CRVE) equivalent^5^. The arterio-venous ratio (AVR) is then calculated by dividing CRAE by CRVE. SVA has been used for cardiovascular risk stratification for the last two decades, primarily in population-based studies. Over 10,000 people were included in the ARIC study (Atherosclerosis Risk in Communities) and results showed a predictive value of narrow arterioles and wide venules for the endpoints mortality, stroke and coronary heart disease^5,6^. Dynamic retinal vessel analysis (DVA) furthermore allows functional assessment of the microcirculation. By applying flickering light to the retina, vessels physiologically dilate^7^. This phenomenon is presumably caused by nitric oxide release and mediated by neurovascular coupling depending on the local metabolic demand in the retina^8^. DVA quantifies this dilation of vessels and calculates parameters representing the percentage of the baseline diameter of the arteriole (aMax) and venule (vMax)^9^. Impaired arteriolar dilation has been described in patients with diabetes mellitus, coronary heart disease and heart failure^10–12^.

Recently, we were able to show a predictive value of DVA parameters for all-cause mortality and cardiovascular mortality in hemodialysis patients^9,13^. However, a direct comparison to individuals without chronic kidney disease and the burden of cardiovascular complications of hemodialysis patients is currently unavailable. Therefore, we examined static and dynamic retinal vessel analysis in 220 individuals without chronic kidney disease or cardiovascular comorbidities and compared them to a cohort of hemodialysis patients.

## Methods

### Study population

Hemodialysis patients were included as part of the “r**IS**k str**A**tification in end-stage **R**enal disease” (ISAR) study (ClinicalTrials.gov: NCT01152892)^14^. A detailed description of the cohort, including the retinal vessel analysis subcohort has already been published in our previous reports^9,13^. Inclusion criteria for patients were current hemodialysis treatment for more than 90 days, age at least 18 years and written and informed consent. Exclusion criteria were malignant disease, current infection, pregnancy and lack of written consent. The patients were recruited in dialysis centers in the greater Munich area.

Inclusion criterion for healthy individuals was age of at least 18 years. Exclusion criteria were chronic or acute kidney disease, diabetes mellitus, heart failure, coronary heart disease including myocardial infarction, transient ischemic attack, history of stroke, cardiac valve dysfunction, untreated arterial hypertension, current infection, chronic infectious diseases (HIV, hepatitis, tuberculosis), epilepsy, glaucoma, degenerative retinal diseases, pregnancy or lack of written/informed consent. Hyperlipidemia and treated arterial hypertension were not part of the exclusion criteria. However, individuals receiving vasodilators and calcium-channel inhibitors were excluded, because of a potential interaction with dilation of vessels. Healthy individuals were part of a control group for the “Citrate-Acetate Study” (ClinicalTrials.gov: NCT02745340), a single-center study investigating the effect of citrate and acetate containing dialysates of hemodialysis patients on the immune phenotype. However, the hemodialysis patients of the “Citrate-Acetate Study” did not receive retinal vessel analysis and are therefore not included in this publication.

All study participants gave written and informed consent. Both studies were performed in accordance to the standards of the 2013 Helsinki Declaration. Additionally, the studies were approved by the local ethics committee (Ethics Committee of the Klinikum rechts der Isar of the Technical University Munich).

### Clinical parameters and comorbidities

Comorbidities and laboratory parameters of hemodialysis patients were assessed as previously described^9^. Information on age, gender, body mass index, smoking status, comorbidities and medications of the healthy individuals were gathered in interviews on the day of the examination. Potential exclusion criteria were screened beforehand via telephone interviews. Treated arterial hypertension was defined as having at least one antihypertensive medication. Similar to the hemodialysis patients, blood pressure measurements were performed with a Mobil-o-Graph monitor (I.E.M., Germany) before retinal vessel analysis. High-sensitivity C-reactive protein (hsCRP) and interleukin-6 (IL-6) were analyzed as previously described. hsCRP is displayed as mg/dl^15^.

### Retinal vessel analysis

All retinal vessel measurements were performed under the same standardized conditions in a room with reduced brightness after 10 min of rest. In case of the dialysis patients, examination was performed before a midweek hemodialysis session. After pupil dilation with topical tropicamide (0.5% Mydriaticum Stulln, Pharma Stulln, Germany) SVA was performed, followed by DVA. In order to enhance patient compliance, the dominant eye was used, if possible. SVA included a series of photographic images of the retina centered to the optic disc. We used a Static Vessel Analyzer (TRC-NW8, Topcon, Tokyo, Japan) taking images at an angle of 50° from one eye. Afterwards retinal arterial (CRAE) and venous (CRVE) equivalents were calculated using the Paar-Hubbard formula^16^ with the standardized software VesselMap (V3.60, Imedos Systems, Jena, Germany). CRAE and CRVE were measured in measuring units (MU). One measuring unit of the imaging device represents 1 µm in Gullstrand’s normal eye model. Arterio-venous ratio (AVR) was calculated as CRAE divided by CRVE. Whenever more than one high-quality image was available, mean CRAE, CRVE and AVR were calculated from a maximum of 3 images. Patients or healthy individuals without at least one high-quality photograph were excluded from the analysis.

Shortly after SVA, DVA measurement was performed on the same eye with a Dynamic Vessel Analyzer (Imedos Systems, Jena, Germany). As previously described, diameters of one arteriole and one venule approximately two disc diameters away from the optic disc were continuously measured (Supplementary Video S1)^9^. 50 s of baseline measurement was followed by three cycles of 20 s lasting flickering light, each intercepted by 80 s of normal light. This amounted to a total measurement time of 350 s. Key parameters of DVA, maximal relative arteriolar dilation in response to flicker (aMax, % to baseline) and maximal relative venular dilation in response to flicker (vMax, % to baseline) were calculated for each study participant as described previously^9^. Quality of the measurements was graded on a scale from zero to five by the first observer, as previously described^17^. Whenever the quality score was below 2.5 the measurement was excluded after discussion with a second observer^9^.

### Statistical analysis

**Figure 1:**
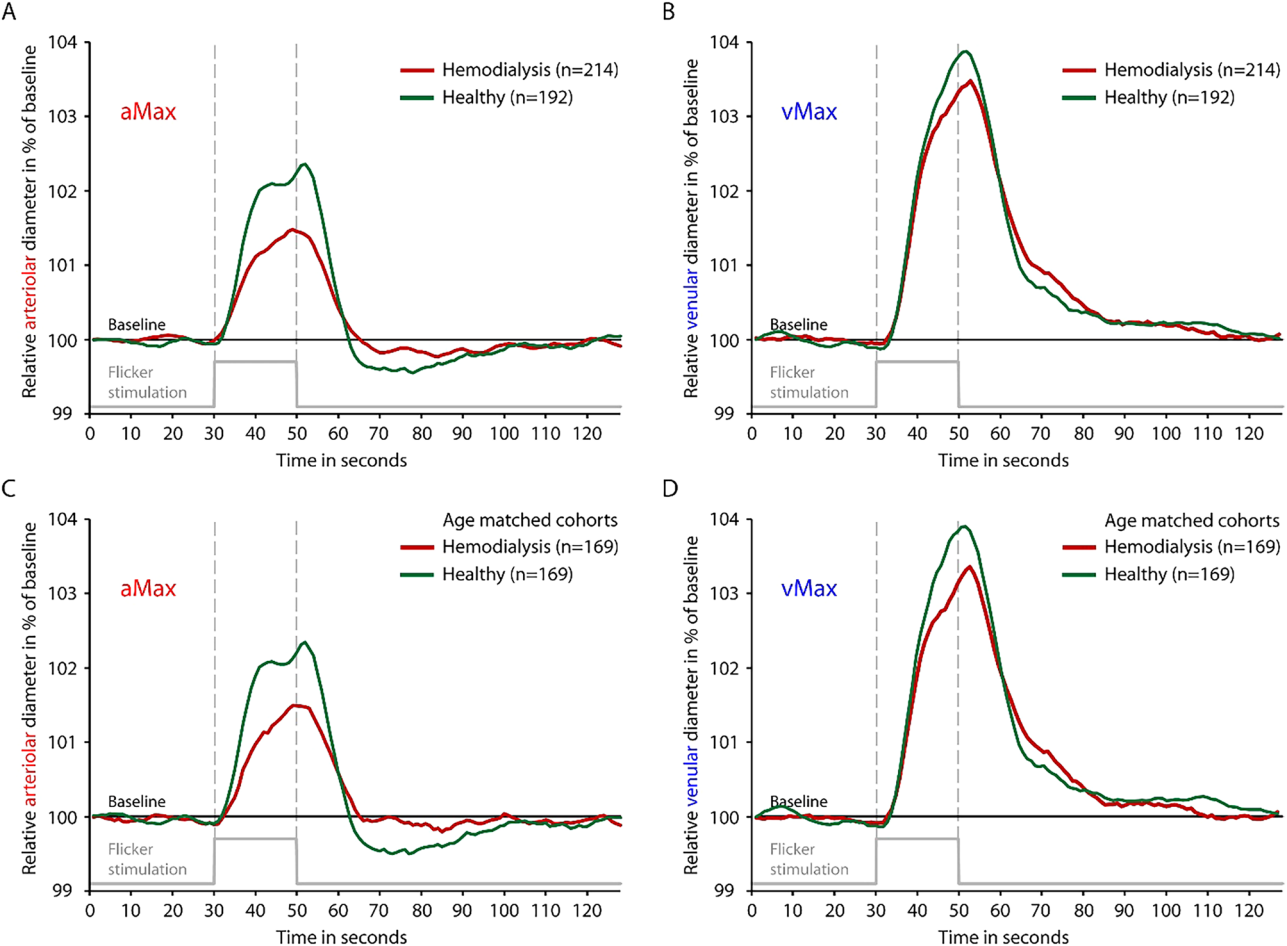
Comparison of time-diameter curves for dialysis patients (red) and healthy individuals (green) for whole cohorts (**A**,**B**) and age-matched cohorts (**C**,**D**). Maximum arteriolar dilation (aMax; **A**,**C**) and maximum venular dilation (vMax; **B**,**D**) were determined near the end of the flickering stimulation as previously described. Light grey line on the bottom of each panel indicates time profile of 20 s lasting flickering light stimulation.

Characteristics of patients and healthy individuals are displayed as mean ± standard deviation for normally distributed variables and as median [interquartile range] for variables following a skewed distribution. Student’s *t*-test was used for comparison between two groups for normal, and Mann–Whitney U test for non-normal data. Categorical data were compared using Chi-square tests (Fig. 1). Correlation analyses were performed with Spearman’s rank correlation coefficient (rho). We performed a case–control matching for age regarding the participants with valid DVA. For participants with valid SVA data a gender and age matching was calculated. As there is no evidence—to our knowledge—for relevant gender-specific differences in DVA, matching was only executed regarding age for the sake of not excluding too many patients/probands. Additionally, gender was not associated with aMax or vMax in a statistically significant fashion in any of the whole or matched DVA subcohorts (healthy and hemodialysis). Case–control matching was performed using fuzzy matching with a tolerance for age matching of 5 years. IBM SPSS Statistics, version 26 (IBM Corporation, Armonk, New York) was used. For visualization in a scatter plot (Fig. 4) and for fitting the linear regression model (Supplementary Table S2) hsCRP was ln-transformed due to the skewed distribution. For graphical demonstration purposes (Fig. 4) a linear regression line was plotted for the univariate relationships between age/hsCRP and DVA parameters aMax and vMax.

## Results

### Demographics of healthy individuals and hemodialysis patients

**Figure 2:**
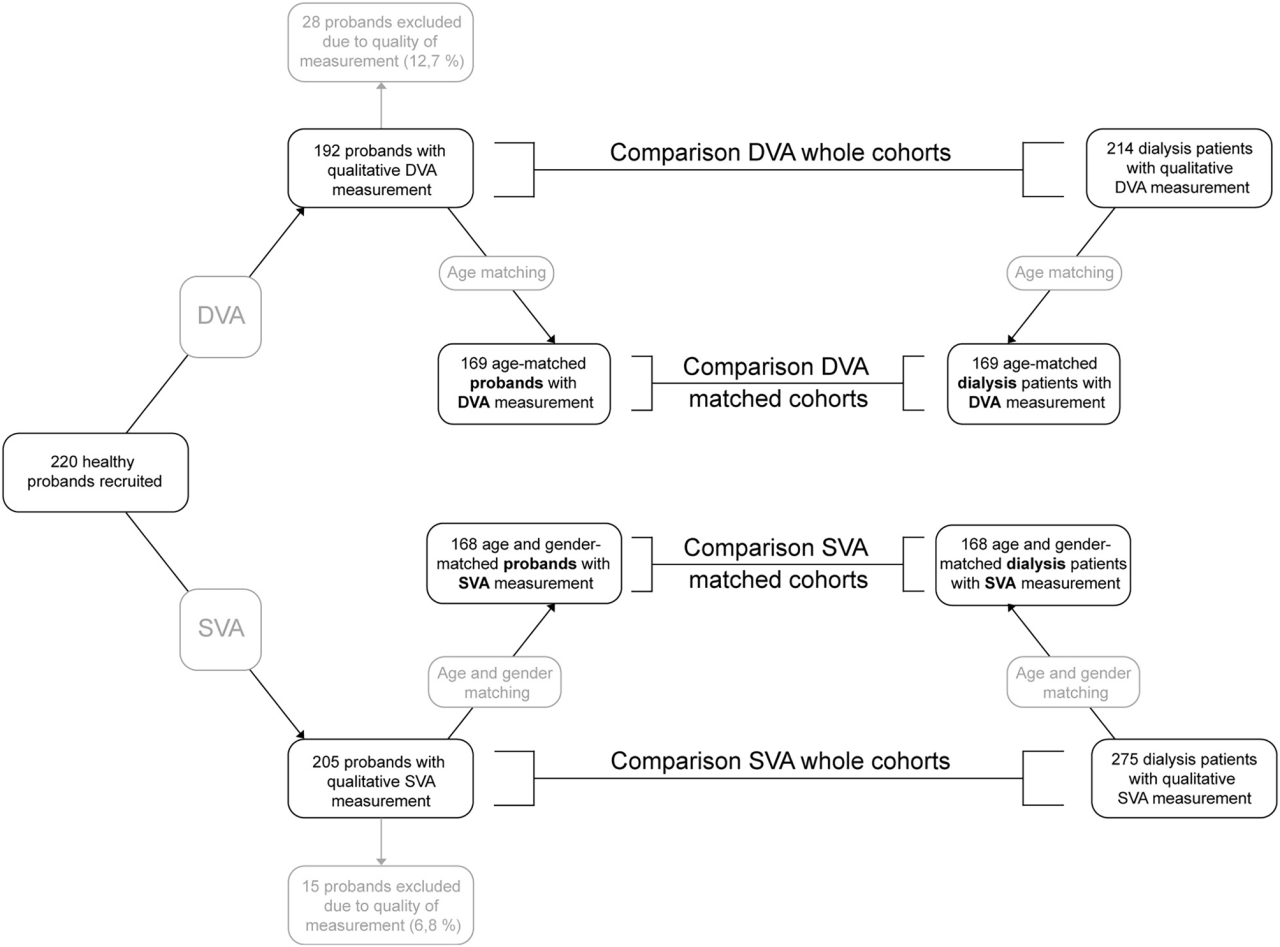
Flowchart illustrating the recruitment of healthy individuals (left side) and the comparison to end-stage renal disease patients (right side). *DVA* dynamic retinal vessel analysis, *SVA* static retinal vessel analysis.

A total of 220 healthy individuals was recruited. After quality evaluation 192 individuals with high-quality DVA measurements (12.7% exclusion) and 205 individuals with high-quality SVA measurement (6.8% exclusion) were available for analysis (Fig. 2). Healthy individuals were on average 65.6 ± 12.5 and 65.8 ± 12.9 years old, in the DVA and SVA subgroup, respectively. The cohort of healthy individuals had a normal mean body mass index and had more female participants (58.3% and 56.6%). Arterial hypertension was present in 18.8% and 20.5% of healthy individuals, regarding DVA and SVA. About every fifth individual had hyperlipidemia and reported an active nicotine abuse. For the hemodialysis patients, 214 patients with high-quality DVA data and 275 patients with high-quality SVA data were available. The characteristics of the DVA subgroup was already described in detail in our previous publications^9,18^ (Table 1). The SVA subgroup was 63.3 ± 14.9 years old and mainly male (71.3%). Patients were slightly overweight (BMI 26.1 ± 5.4 kg/m^2^), 32.4% had diabetes mellitus and 53.1% vascular comorbidities including coronary heart disease, peripheral vascular disease, history of cerebrovascular events and arteriosclerosis (Supplementary Table S1).

**Table 1:** Characteristics of included dialysis patients and healthy individuals as well as age-matched cohort for DVA.

	Whole cohorts		p-value	Age-matched cohorts for DVA		p-value
	Healthy	Hemodialysis		Healthy	Hemodialysis	
n	192	214	–	169	169	–
Age, years, mean ± SD	65.6 ± 12.5	62.6 ± 15.0	0.030	65.4 ± 12.7	65.4 ± 13.1	0.996
BMI, kg/m^2^, mean ± SD	24.4 ± 3.6	26.1 ± 5.5	< 0.001	24.5 ± 3.6	26.4 ± 5.4	< 0.001
Gender, male in %	41.7%	68.2%	< 0.001	40.2%	67.5%	< 0.001
Systolic blood pressure, mmHg, mean ± SD	132 ± 15	134 ± 22	0.252	132 ± 16	135 ± 22	0.219
Diastolic blood pressure, mmHg, mean ± SD	84 ± 10	74 ± 14	< 0.001	84 ± 10	74 ± 15	< 0.001
Mean blood pressure, mmHg, mean ± SD	105 ± 11	94 ± 15	< 0.001	106 ± 11	94 ± 15	< 0.001
eGFR (mean ± SD; CKD-EPI 2009; ml/min/1.73 m^2^)	76 ± 16	–	–	77 ± 15	–	–
Vascular disease*	0%	47.7%	–	0%	53.8%	–
Diabetes mellitus	0%	30.8%	–	0%	35.5%	–
Dialysis vintage in months, median [IQR]	–	48.5 [24.0–79.0]	–	–	42.0 [21.0–76.0]	–
Arterial hypertension in %	18.8%	94.4%	< 0.001	20.7%	94.5%	< 0.001
Hyperlipidemia in %	17.2%	57.9%	< 0.001	17.8%	64.5%	< 0.001
Nicotine abuse in %	20.3%	23.2%	0.494	20.7%	21.4%	0.881
Calcium channel blocker in medication in %	–	35.0%	–	–	36.1%	–
Vasodilator in medication in %	–	13.6%	–	–	14.2%	–
hsCRP, mg/dl, median [IQR]	0.13 [0.06–0.26]	0.36 [0.18–0.81]	< 0.001	0.13 [0.06–0.26]	0.39 [0.19–0.92]	< 0.001

*Including coronary heart disease, peripheral artery disease, history of cerebrovascular events, arteriosclerosis.

*BMI* body mass index, *hsCRP* high-sensitivity C-reactive protein, *eGFR* estimated glomerular filtration rate.

Comparing healthy individuals with patients on hemodialysis, patients were significantly older, had a higher BMI, more often arterial hypertension and hyperlipidemia, and had lower diastolic and mean arterial blood pressure. Additionally, hemodialysis patients exhibited enhanced systemic inflammation as illustrated by median hsCRP parameters (0.36 vs 0.13 mg/dl).

### Retinal endothelial dysfunction in dialysis patients

**Figure 3:**
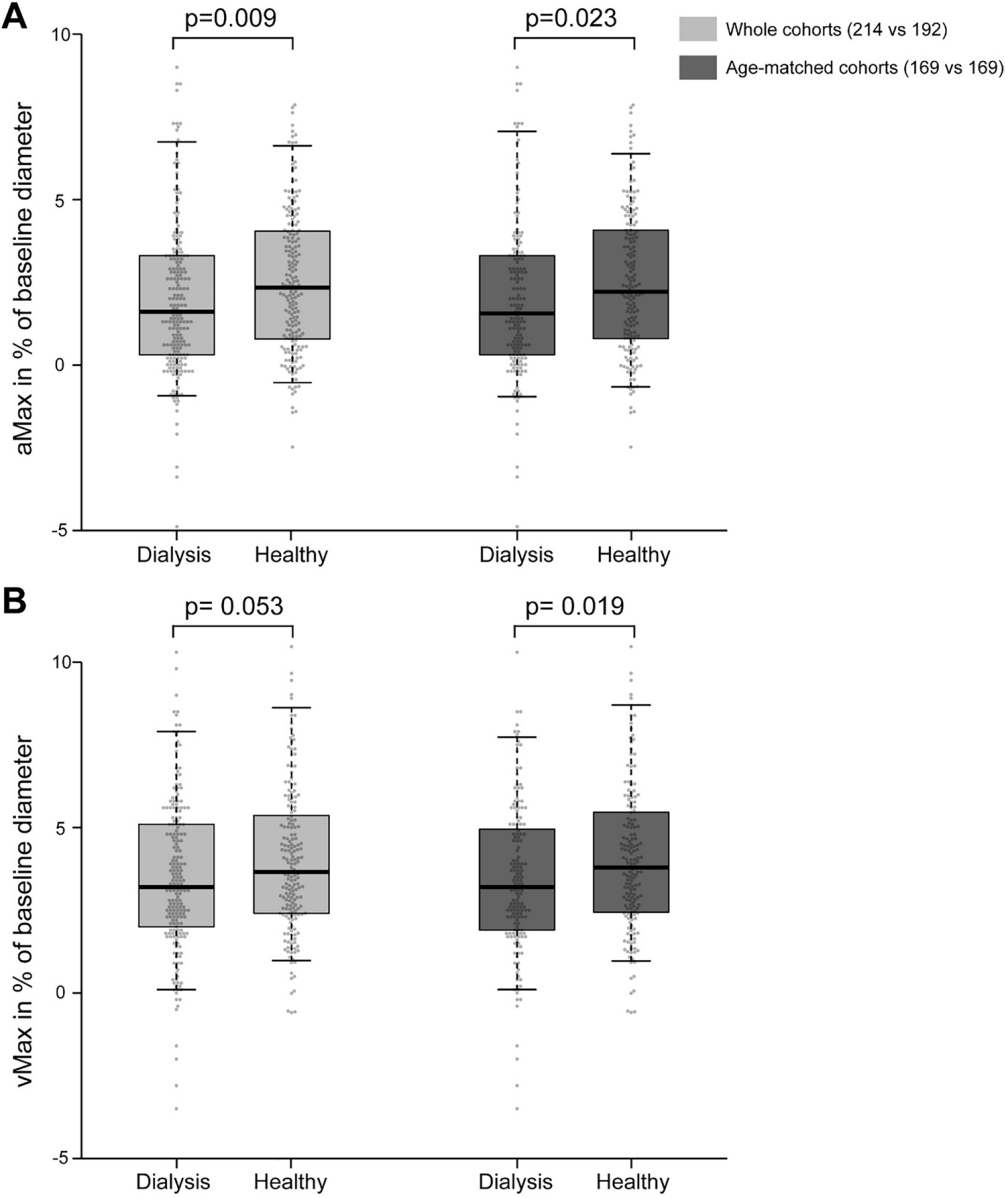
Comparison of maximum arteriolar dilation (aMax; **A**) and maximum venular dilation (vMax; **B**) between dialysis patients and healthy individuals. Boxplots are displayed for whole cohorts (grey bars, left side) and age-matched cohorts (darker bars, right side). Horizontal line represents median. Box represents interquartile range. Whiskers represent 95% confidence interval of mean. Data points are overlayed transparently.

Hemodialysis patients showed reduced arteriolar and venular vasodilation compared to healthy individuals, as illustrated in time-diameter curves (Fig. 1). To quantify those differences, aMax significantly differed (p = 0.009) between the two groups with 1.6 [0.3–3.3]% dilation above baseline diameter for dialysis patients and 2.3 [0.7–4.1]% above baseline for healthy individuals (Fig. 3). vMax also showed reduced, but not significantly different, dilation in the dialysis group (3.2 [2.0–5.1]% vs. 3.7 [2.4–5.4]%, p = 0.053). Due to the importance of age as a confounder of DVA, we executed a case–control matching resulting in 169 cases vs 169 controls with a mean age of 65.4 years. The remaining characteristics of the cohorts did not relevantly change (Table 1). Comparison of the matched cohorts show a similar difference in aMax and vMax for the two groups. Both differences showed statistical significance (aMax p = 0.023; vMax p = 0.019; Table 4).

### Arteriolar retinal dilation in healthy subjects depends on age with a potential role for systemic inflammation

**Figure 4:**
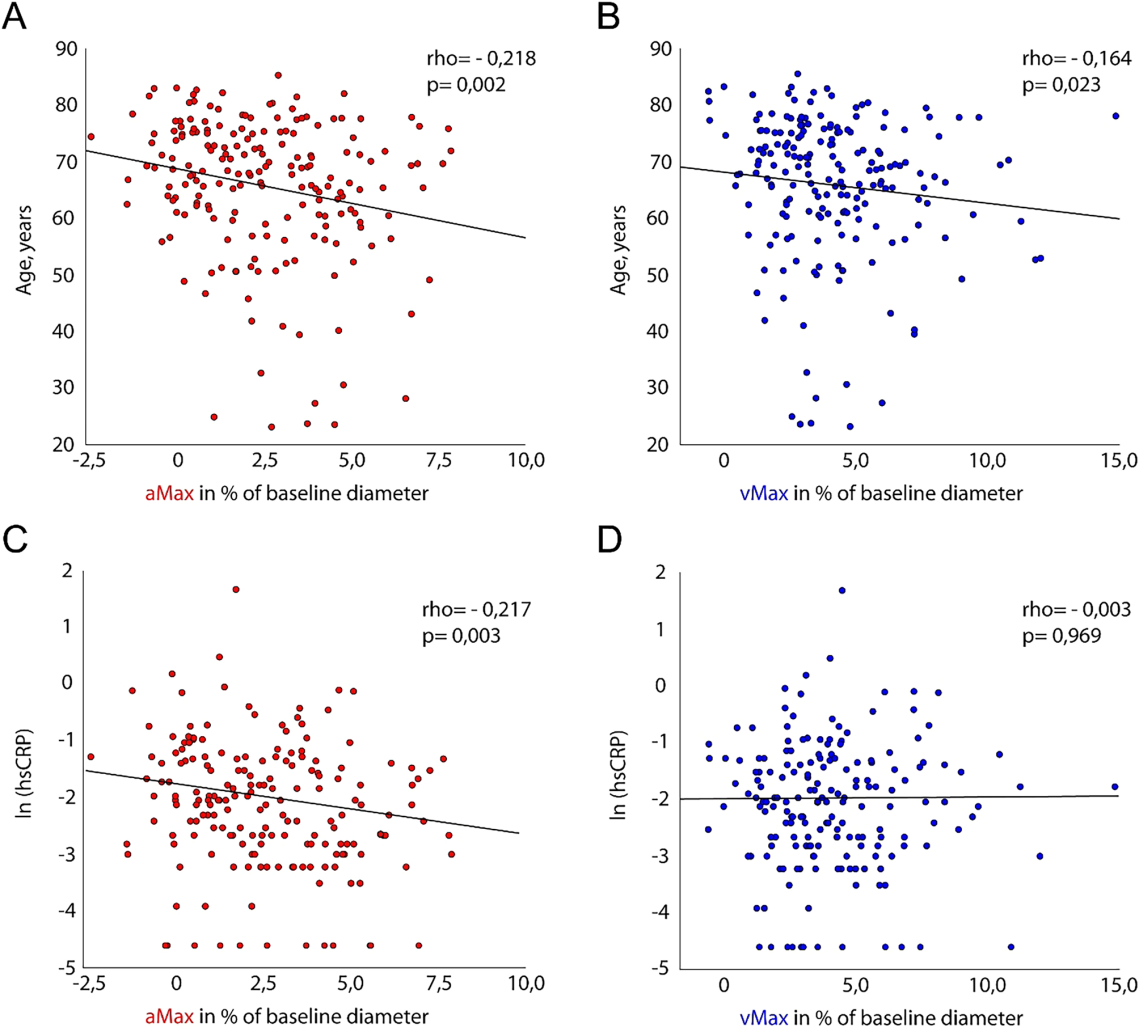
Associations of aMax and vMax with age and hsCRP in healthy individuals. Scatter-dot plots show maximum arteriolar dilation (aMax; red; **A**,**C**) and maximum venular dilation (vMax; blue; **B**,**D**). Spearman’s rho is displayed for each association. hsCRP was ln-transformed due to non-normally distributed data. Linear regression lines were plotted for illustrational purposes.

The primary DVA parameters aMax and vMax showed a negative association with age in healthy individuals (Table 2). Thus, flicker-induced dilation of arterioles and venules decreases with older age. This association was statistically significant for both parameters. However, a stronger association was observed for aMax (Fig. 4). Interestingly, the systemic inflammation parameter hsCRP was negatively associated with aMax, but not vMax. The relationship of aMax with hsCRP was weak, yet highly significant (rho = − 0.217; p = 0.003). After ln-transformation of hsCRP (Fig. 4C,D) for linear regression analysis and adjustment for age, the association with aMax was reduced and the statistical significance was lost (Supplementary Table S2; beta = − 0.134; p = 0.068).

**Table 2:** Correlation of DVA parameters with healthy individuals’ characteristics (n = 192).

	aMax	p-value*	vMax	p-value*
Age, years	**− 0.218**	**0.002**	**− 0.164**	**0.023**
BMI, kg/m^2^	− 0.019	0.795	0.017	0.813
Gender, male	− 0.019	0.792	− 0.122	0.091
Systolic blood pressure, mmHg	− 0.080	0.271	0.062	0.393
Diastolic blood pressure, mmHg	0.020	0.783	0.115	0.115
Mean blood pressure, mmHg	− 0.030	0.684	0.113	0.119
Arterial hypertension	− 0.010	0.889	− 0.057	0.434
Hyperlipidemia	− 0.009	0.900	0.063	0.386
Nicotine abuse	− 0.050	0.900	− 0.029	0.691
hsCRP, mg/dl	− **0.217**	**0.003**	− 0.003	0.969

*BMI* body mass index, *hsCRP* high-sensitivity C-reactive protein, *aMax* maximum arteriolar dilation, *vMax* maximum venular dilation.

*Significant correlations are marked bold (p < 0.05).

Hemodialysis patients showed a significant negative association of vMax with age (rho = − 0.201, p = 0.003; Supplementary Table S3). aMax was not statistically significant associated with age. Dialysis vintage and medication with calcium channel blockers or vasodilators did not associate with aMax or vMax. However, diabetic patients showed reduced aMax, but not vMax compared to non-diabetic patients (Supplementary Table S5).

hsCRP and IL-6 correlated with reduced dilation of retinal vessels, but only for venules (rho = − 0.180 and − 0.246, p = 0.011 and < 0.001). The association of aMax and hsCRP as well as aMax and IL-6 showed a weak and non-significant trend (Supplementary Table S3).

### SVA parameters showed no differences between healthy individuals and hemodialysis patients

Static vessel diameters showed various associations with age, blood pressure and nicotine abuse. Older individuals typically had narrower arterioles (Table 3). Higher systolic, diastolic and mean arterial pressure were associated with narrower arterioles as well as venules in healthy individuals. This association was more pronounced for arterioles, leading to a lower AVR in patients with high blood pressure. Furthermore, nicotine abuse resulted in significantly wider retinal arterioles and venules. In our cohort, gender was not associated with measurements of SVA (Table 3).

**Table 3:** Correlation of SVA parameters with characteristics of healthy individuals (n = 205).

	CRAE	p-value*	CRVE	p-value*	AVR	p-value*
Age, years	**− 0.209**	**0.003**	− 0.006	0.933	**− 0.244**	** < 0.001**
BMI, kg/m^2^	− 0.029	0.679	0.014	0.845	**− 0.138**	**0.048**
Gender, male	0.092	0.191	− 0.009	0.898	0.098	0.161
Systolic blood pressure, mmHg	**− 0.327**	** < 0.001**	**− 0.147**	**0.036**	**− 0.215**	**0.002**
Diastolic blood pressure, mmHg	**− 0.225**	**0.001**	**− 0.174**	**0.013**	**− 0.143**	**0.042**
Mean blood pressure, mmHg	**− 0.282**	** < 0.001**	**− 0.174**	**0.013**	**− 0.169**	**0.016**
Arterial hypertension	− 0.066	0.344	− 0.051	0.465	0.001	0.998
Hyperlipidemia	0.011	0.876	0.054	0.444	− 0.057	0.416
Nicotine abuse	**0.173**	**0.013**	**0.183**	**0.009**	− 0.024	0.735
hsCRP, mg/dl	0.005	0.939	0.068	0.340	− 0.049	0.497

*BMI* body mass index, *hsCRP* high-sensitivity C-reactive protein, *CRAE* central retinal arterial equivalent, *CRVE* central retinal venous equivalent, *AVR* arterio-venous ratio.

*Significant correlations are marked bold (p < 0.05).

Hemodialysis patients showed a similar pattern with significantly narrower arterioles in patients with higher systolic and mean blood pressure. Interestingly, CRVE did not associate with blood pressure measurements in hemodialysis patients (Supplementary Table S4). Diabetic patients showed significantly reduced CRAE, but not CRVE, compared to non-diabetic patients (Supplementary Table S6). Dialysis vintage was not associated with SVA parameters.

Comparing SVA parameters between healthy individuals and hemodialysis patients showed no significant difference (Table 4). As gender-specific differences for those parameters have been described, we performed a case–control matching for gender. The resulting subgroups included 168 cases and 168 controls with no statistically significant differences in age and gender (Supplementary Table S1). However, SVA parameters CRAE, CRVE und AVR still did not show any differences between hemodialysis patients and healthy controls.

**Table 4:** Comparison of SVA parameters and additional parameters of DVA between dialysis patients and healthy individuals.

	Whole cohorts			Matched cohorts^§^		
	Healthy*	Hemodialysis*	p-value†	Healthy*	Hemodialysis*	p-value†
aMax, %	**2.3 [0.7–4.1]**	**1.6 [0.3–3.3]**	**0.009**	**2.2 [0.8–4.1]**	**1.6 [0.3–3.3]**	**0.023**
vMax, %	3.7 [2.4–5.4]	3.2 [2.0–5.1]	0.053	**3.8 [2.4–5.5]**	**3.2 [1.9–5.1]**	**0.019**
CRAE, MU	175 [165–185]	176 [165–185]	0.998	176 [165–185]	176 [164–186]	0.838
CRVE, MU	209 [196–220]	208 [196–220]	0.667	208 [195–220]	209 [196–223]	0.534
AVR	0.84 [0.80–0.88]	0.84 [0.80–0.89]	0.703	0.84 [0.81–0.88]	0.84 [0.79–0.88]	0.589

*aMax* maximum arteriolar dilation, *vMax* maximum venular dilation, *CRAE* central retinal arterial equivalent, *CRVE* central retinal venous equivalent, *AVR* arterio-venous ratio.

^†^Significant differences are marked bold (p < 0.05).

*Data displayed as median [IQR].

^§^Matching for DVA parameters for age (n = 169 vs 169); matching for SVA parameters for age and gender (n = 168 vs 168).

## Discussion

In this first study comparing retinal microvascular dysfunction in hemodialysis patients and age-matched healthy controls, we found a significant attenuation of arteriolar dilation in hemodialysis patients. For venular dilation a smaller, yet significant difference could be detected. SVA parameters however did not significantly differ between the two groups. The latter observation might surprise, as hemodialysis patients exhibited a lot of vascular comorbidities. In a recent publication Streese et al. described age and blood pressure as the main determinants of narrowing of arterioles and venules in healthy individuals^7^. According to the standardized age-SVA diagrams, a 65-year-old healthy individual has a median CRAE of 174 MU, CRVE of 206 MU and an AVR of 0.84. Those parameters are similar to both our cohorts. It remains to be elucidated in future studies, whether the finding may be due to the extensive calcification typically seen in these patients. Once hemodialysis is initiated, vascular smooth muscle cells show increased calcification and apoptosis^19^, which might lead to increased vessel diameters to an extent whereby arterioles are comparable to the diameters in healthy individuals with normal endothelial function. Nonetheless, our data seem to suggest, that in cohorts of multimorbid patients like with end-stage renal disease, SVA might not give a lot of additional information regarding the microvascular damage, before and after matching for gender and age.

In comparison, DVA showed reduced aMax in dialysis patients compared to controls. Age has previously been described in the literature as an important factor influencing arteriolar dilation^20^. After adjusting for age in our study, dialysis patients still showed significantly reduced aMax. While a difference to healthy probands of 0.7% in aMax might seem small, its impact on volume blood flow is magnified due to the fourth power calculation of radius in Pouseuille’s law. Our hemodialysis patients expectedly had many comorbidities including diabetes mellitus (30.8%) and vascular diseases (47.7%). Coronary heart disease, heart failure and diabetes mellitus have already been described as diseases, that reduce arteriolar dilation as a marker of endothelial dysfunction^10–12^. The discrepancy between DVA and SVA parameters emphasizes, that in multimorbid patients DVA might give a more precise picture of microvascular damage. Longitudinal studies are required for frequent diseases like diabetes mellitus and coronary heart disease, to not only show a difference in comparison to healthy controls, but to prove a predictive effect of DVA parameters on clinical outcome. Other methods for measuring endothelial dysfunction like flow-mediated dilation should also be included in future studies to determine an optimal biomarker for endothelial dysfunction. In our study, venular dilation was also impaired in hemodialysis patients compared to controls albeit less than for arteriolar dilation. This is surprising, because vMax has been identified as an independent predictor for mortality in our (same) cohort of hemodialysis patients^9^. aMax on the other hand, did not predict mortality, but was only able to (weakly and not independently) predict cardiovascular events. One can only speculate why aMax shows a bigger difference than vMax in comparison with healthy patients, while vMax has the best predictive value. A possible hypothesis is, that hemodialysis patients suffer extensive vascular damage within the arteriolar bed (vascular calcification, severe hypertension), which is worse than in the venular vessels. Retinal vessel dysfunction may be evident at a later more severe disease state characterized by advanced inflammation. Once venular dysfunction can be diagnosed, this appears to affect estimates of mortality^9,18^. The two major dangers for hemodialysis patients are cardiovascular and infection-associated mortality, which are both driven by chronic inflammation^20^. The systemic inflammation parameters hsCRP and IL-6 were negatively associated with venular dilation in our hemodialysis cohort. On the contrary, in healthy individuals hsCRP was negatively associated with aMax, but not vMax. However, the latter association of aMax and hsCRP was diminished after adjustment for age. Systemic inflammation is one of the main factors leading to endothelial dysfunction^3^. The exact mechanisms behind flicker-induced vasodilation still have to be revealed. The question remains as to why inflammation in hemodialysis patients only associates with decreased venular dilation, but not arteriolar dilation. Throughout the last years DVA has been adjusted to be used in rodents. Only recently a study was able to prove an involvement of voltage-gated calcium channels in knock-out mice^21^. Further in-vivo studies in rodents are needed to clarify the role of inflammation in the pathophysiology of retinal microvascular dysfunction.

The characteristics of the healthy individuals is certainly one limitation of our study. Individuals with hyperlipidemia and treated arterial hypertension were able to take part in the examination. However, the prevalence of arterial hypertension in the general population is approximately 45% and even 75% in individuals over 60 years according to a recent survey of the United States Center for Disease Control and Prevention^22^. For hyperlipidemia, about 56–60% of the German population show moderately elevated cholesterol levels and 18–20% highly elevated cholesterol concentrations^23^. In our cohort with a mean age of 65 years 17% had hyperlipidemia and only 19% of patients had arterial hypertension, which is far below the expected prevalence. The aim of our study was to compare hemodialysis patients to age-matched individuals of the general population without chronic kidney disease and without cardiovascular complications. The difference in aMax and vMax between those two cohorts is rather small. As a consequence of the mean age of our healthy cohort, it certainly includes some individuals with cardiovascular risk, which might diminish the difference in aMax and vMax between the cohorts. It is possible that a comparison to individuals in perfect health might have turned out different results. However, given the convincing data showing the value of vMax for mortality prediction^9^, it seems DVA might be more useful for risk stratification than for differing between dialysis patients and healthy individuals.

Another limitation involves the method of retinal vessel analysis and the requirement of patient compliance for DVA. Patients and individuals needed to be able to follow the examiner’s instructions and non-adherence can result in exclusion of the patient due to insufficient image quality. The multimorbidity of hemodialysis patients leads to a high exclusion rate (about 25% with insufficient image quality for DVA). Hemodialysis patients with sufficient DVA quality were therefore slightly healthier than the average dialysis patients^9,24^. A comparison to more multimorbid hemodialysis patients could have shown different results for DVA. Nonetheless, following the hypothesis of attenuated aMax and vMax in dialysis patients, the difference to the healthy individuals might have even been more emphasized in more multimorbid patients. This is underlined by the fact, that diabetic patients with increased comorbidities showed more attenuated vasodilation. Additionally, given the artificial nature of flickering light, there’s a possibility that more physiological stimuli could result in larger diameter adaptions of vessels. In conclusion, our study provides first evidence of diminished retinal arteriolar and venular microvascular function in hemodialysis patients compared to healthy controls. A relevant difference for static arteriolar and venular vessel diameters was absent in the same two cohorts. DVA seems to be able to identify the damaged microvasculature better than SVA. Therefore, further studies on retinal endothelial dysfunction in multimorbid patients should shift their focus on DVA to determine biomarkers and risk factors for microvascular health.

### Supplementary Information

Supplementary Tables.

Supplementary Video 1.

## Data Availability

The data underlying this article will be shared on reasonable request to the corresponding author.

## References

[1] Cozzolino M (2018). Cardiovascular disease in dialysis patients. Nephrol. Dial. Transplant..

[2] Bernelot Moens SJ (2017). Arterial and cellular inflammation in patients with CKD. J. Am. Soc. Nephrol..

[3] Roumeliotis S, Mallamaci F, Zoccali C (2020). Endothelial dysfunction in chronic kidney disease, from biology to clinical outcomes: A 2020 update. J. Clin. Med..

[4] Deanfield JE, Halcox JP, Rabelink TJ (2007). Endothelial function and dysfunction: Testing and clinical relevance. Circulation.

[5] Seidelmann SB (2016). Retinal vessel calibers in predicting long-term cardiovascular outcomes: The atherosclerosis risk in communities study. Circulation.

[6] Yatsuya H (2010). Retinal microvascular abnormalities and risk of lacunar stroke: Atherosclerosis risk in communities study. Stroke J. Cereb. Circ..

[7] Streese L (2021). Normative data and standard operating procedures for static and dynamic retinal vessel analysis as biomarker for cardiovascular risk. Sci. Rep..

[8] Dorner GT (2003). Nitric oxide regulates retinal vascular tone in humans. Am. J. Physiol. Heart Circ. Physiol..

[9] Gunthner R (2019). Impaired retinal vessel dilation predicts mortality in end-stage renal disease. Circ. Res..

[10] Barthelmes J (2019). Retinal microvascular dysfunction in patients with coronary artery disease with and without heart failure: A continuum?. Eur. J. Heart Fail..

[11] Nagele MP (2018). Retinal microvascular dysfunction in heart failure. Eur. Heart J..

[12] Sorensen BM (2016). Prediabetes and type 2 diabetes are associated with generalized microvascular dysfunction: The Maastricht study. Circulation.

[13] Werfel S (2021). Identification of cardiovascular high risk groups from dynamic retinal vessel signals using untargeted machine learning. Cardiovasc. Res..

[14] Schmaderer C (2016). Rationale and study design of the prospective, longitudinal, observational cohort study "rISk strAtification in end-stage renal disease" (ISAR) study. BMC Nephrol.

[15] Lorenz G (2018). Mortality prediction in stable hemodialysis patients is refined by YKL-40, a 40-kDa glycoprotein associated with inflammation. Kidney Int..

[16] Hubbard LD (1999). Methods for evaluation of retinal microvascular abnormalities associated with hypertension/sclerosis in the Atherosclerosis Risk in Communities Study. Ophthalmology.

[17] Kotliar K (2017). Altered neurovascular coupling as measured by optical imaging: A biomarker for Alzheimer's disease. Sci. Rep..

[18] Gunthner R (2022). Mortality prediction of retinal vessel diameters and function in a long-term follow-up of haemodialysis patients. Cardiovasc. Res..

[19] Shroff RC (2008). Dialysis accelerates medial vascular calcification in part by triggering smooth muscle cell apoptosis. Circulation.

[20] Seshadri S, Ekart A, Gherghel D (2016). Ageing effect on flicker-induced diameter changes in retinal microvessels of healthy individuals. Acta Ophthalmol..

[21] Neumaier F (2021). Retinal vessel responses to flicker stimulation are impaired in Ca v 23-deficient mice-an in-vivo evaluation using retinal vessel analysis (RVA). Front. Neurol..

[22] Ostchega, Y., Fryar, C. D., Nwankwo, T. & Nguyen, D. T. Hypertension prevalence among adults aged 18 and over: United States, 2017–2018. *NCHS Data Brief* 1–8 (2020). 32487290

[23] Scheidt-Nave C (2013). Prevalence of dyslipidemia among adults in Germany: Results of the German Health Interview and Examination Survey for Adults (DEGS 1). Bundesgesundheitsblatt, Gesundheitsforschung, Gesundheitsschutz.

[24] Saran R (2017). US renal data system 2016 annual data report: Epidemiology of kidney disease in the United States. Am. J. Kidney Dis. Off. J. Natl. Kidney Found..

