# Supplementary material for: Endothelial dysfunction in retinal vessels of hemodialysis patients compared to healthy controls
Source: Sci Rep. 2024 Jun 17;14:13948. doi: 10.1038/s41598-024-64581-9 (PMC11183144; doi:10.1038/s41598-024-64581-9)
Supplement: Supplementary file 1 — Supplementary Tables. [file 41598_2024_64581_MOESM1_ESM.pdf]

Supplementary material

Endothelial dysfunction in retinal vessels of hemodialysis patients compared to healthy controls

Roman Günthner, Georg Lorenz, Matthias Christoph Braunisch, Susanne Angermann, Julia Matschkal, Renate Hausinger, Timon Kuchler, Patrizia Glaser, Felix Schicktanz, Bernhard Haller, Uwe Heemann, Lukas Streese, Henner Hanssen, Konstantin Kotliar, Christoph Schmaderer

Supplementary Table 1. Characteristics of included dialysis patients and healthy individuals for age and gender matched cohort for SVA

	Whole cohort SVA			Age and gender matched cohorts for SVA		
	Healthy	Hemodialysis	p-value	Healthy	Hemodialysis	p-value
n	205	275	-	168	168	-
Age, years, mean ± SD	65.8 ± 12.9	63.3 ± 14.9	0.050	65.4 ± 13.5	63.7 ± 15.1	0.287
BMI, kg/m², mean ± SD	24.5 ± 3.7	26.1 ± 5.4	<0.001	24.8 ± 3.8	25.7 ± 5.8	0.064
Gender, male in %	43.4 %	71.3%	<0.001	53.0 %	53.0 %	-
Systolic blood pressure, mmHg, mean ± SD	132 ± 15	135 ± 22	0.079	133 ± 16	135 ± 24	0.298
Diastolic blood pressure, mmHg, mean ± SD	84 ± 10	73 ± 14	<0.001	84 ± 10	73 ± 16	<0.001
Mean blood pressure, mmHg, mean ± SD	105 ± 11	94 ± 15	<0.001	106 ± 11	94 ± 16	<0.001
eGFR, mean ± SD, ml/min/1.73 m^2	77 ± 16	-	-	78 ± 16	-	-
Vascular disease* in %	-	53.1%	-	0 %	52.4 %	-
Diabetes mellitus in %	-	32.4%	-	0 %	30.4 %	-
Dialysis vintage in months, median [IQR]	-	48.0 [23.0 -81.5]	-	-	52.0 [25.0-85.5]	-
Arterial hypertension in %	20.5 %	93.1%	<0.001	20.8 %	91.1 %	<0.001
Hyperlipidemia in %	17.1%	61.1%	<0.001	18.5 %	61.3 %	<0.001
Nicotine abuse in %	19.9%	24.5%	0.159	20.2 %	23.6 %	0.468
Calcium channel blocker in medication in %	-	35.3 %	-	-	31.0 %	-
Vasodilator in medication in %	-	12.4 %	-	-	8.3 %	-
hsCRP, mg/dl, median [IQR]	0.13 [0.06-0.26]	0.38[0.17-0.88]	<0.001	0.13 [0.06-0.26]	0.34 [0.17-0.81]	<0.001

* including coronary heart disease, peripheral artery disease, history of cerebrovascular events, arteriosclerosis

BMI: body mass index; hsCRP: high-sensitivity C-reactive protein; eGFR: estimated glomerular filtration rate

Supplementary Table 2. Linear regression model for aMax in healthy individuals

Dependent variable aMax	**Model 1***		**Model 2§**	
	Standardized beta	p-value†	Standardized beta	p-value†
ln (hsCRP)	**-0.162**	**0.028**	-0.134	0.068
Age, years	-	-	**-0.181**	**0.014**
R^2	0.026		0.058	

† significant differences are marked bold (p<0.05)
* Model 1 including logarithmic-transformed hsCRP
§ Model 2 including logarithmic-transformed hsCRP and age

hsCRP: high-sensitivity C-reactive protein; aMax: maximum arteriolar dilation

Supplementary Table 3. Correlation of DVA parameters with characteristics of hemodialysis patients (n=214)

	aMax	p-value*	vMax	p-value*
Age, years	-0.124	0.071	**-0.201**	**0.003**
BMI, kg/m²	-0.083	0.229	-0.060	0.384
Gender, male	0.025	0.715	-0.095	0.166
Systolic blood pressure, mmHg	0.017	0.809	0.046	0.500
Diastolic blood pressure, mmHg	0.104	0.128	0.068	0.325
Mean blood pressure, mmHg	0.070	0.308	0.066	0.339
Vascular comorbidity	**-0.194**	**0.005**	**-0.267**	**<0.001**
Diabetes mellitus	**-0.142**	**0.038**	-0.108	0.116
Arterial hypertension	**-0.186**	**0.007**	-0.095	0.167
Hyperlipidemia	-0.082	0.232	**-0.145**	**0.035**
Dialysis vintage	0.026	0.704	0.018	0.790
Nicotine abuse	0.017	0.812	0.023	0.745
hsCRP, mg/dl	-0.101	0.155	**-0.180**	**0.011**
IL-6, pg/ml	-0.050	0.486	**-0.246**	**<0.001**

* significant correlations are marked bold (p<0.05)

BMI: body mass index; hsCRP: high-sensitivity C-reactive protein; aMax: maximum arteriolar dilation; vMax: maximum venular dilation; IL-6: interleukin 6

Supplementary Table 4. Correlation of SVA parameters with blood pressure measurements in hemodialysis patients

	CRAE	p-value*	CRVE	p-value*	AVR	p-value*
Systolic blood pressure, mmHg	**-0.277**	**<0.001**	-0.075	0.216	**-0.273**	**<0.001**
Diastolic blood pressure, mmHg	-0.019	0.755	-0.050	0.405	0.041	0.495
Mean blood pressure, mmHg	**-0.152**	**0.012**	-0.071	0.243	-0.110	0.068

* significant correlations are marked bold (p<0.05)

Supplementary Table 5. Comparison of parameters of DVA between diabetic and non-diabetic hemodialysis patients

	**Hemodialysis diabetic (n=66)**	**Hemodialysis non-diabetic (n=148)**	**p-value**
**Age in years, mean ± SD**	69.8 ± 10.5	59.4 ± 15.6	<0.001
**Vascular disease in %***	68 %	39 %	<0.001
**aMax in %, median [IQR]**	1.1 [0.1 – 2.9]	2.0 [0.4 – 3.3]	0.039
**vMax in %, median [IQR]**	2.6 [1.8 – 4.5]	3.4 [2.1 – 5.3]	0.116

* including coronary heart disease, peripheral artery disease, history of cerebrovascular events, arteriosclerosis

Supplementary Table 6. Comparison of parameters of SVA between diabetic and non-diabetic hemodialysis patients

	**Hemodialysis diabetic (n=89)**	**Hemodialysis non-diabetic (n=186)**	**p-value**
**Age in years, mean ± SD**	71.5 ± 9.9	59.3 ± 15.3	<0.001
**Vascular disease in %***	75 %	42 %	<0.001
**CRAE in MU, mean ± SD**	170 ± 19	177 ± 18	0.004
**CRVE in MU, mean ± SD**	206 ± 21	209 ± 20	0.315

* including coronary heart disease, peripheral artery disease, history of cerebrovascular events, arteriosclerosis
